# Bioaccumulation of Metals in Brain, Eye, Skeleton, and Skin Tissues of Wastewater-Fed Fish: A Case Study in Turkey

**DOI:** 10.3390/toxics14030205

**Published:** 2026-02-27

**Authors:** Aslıhan Katip

**Affiliations:** Department of Environmental Engineering, Engineering Faculty, Bursa Uludağ University, 16059 Bursa, Turkey; aballi@uludag.edu.tr; Tel.: +90-224-294-0918

**Keywords:** bioaccumulation, metals, wastewater-fed fish, tissue-specific metal accumulation, food safety and risk assessment

## Abstract

In this study, the metal accumulation (Cd, Cr, Cu, Fe, Mn, Ni, Pb, and Zn) and toxicity status in the brain, eye, skeleton, and skin tissues of *Carassius gibelio* species of fish fed with wastewater treated with secondary treatment were investigated, and the usability of wastewater and fish for human food against climate change and food crisis was investigated. Treated wastewater (TWW) complied with Turkish aquaculture standards, but was not found to comply with drinking and irrigation water standards. The national and international food standards for metal concentrations varied. Cd and Pb were found to be high in all tissues according to all standards, but Cu, Cr, Mn, Ni, Fe, and Zn were found to vary according to tissues. It was determined that Fe and Zn concentrations were generally higher than those of the other metals in all tissues. The orders of the metals according to their annual mean concentrations were: Zn > Fe > Mn > Pb > Ni > Cr > Cu > Cd in skeletons; Zn > Fe > Pb > Ni > Cr > Cu > Mn > Cd in skins; Zn > Fe > Cu > Pb > Mn > Ni > Cr > Cd in eyes; and Fe > Zn > Pb > Cu > Cr > Ni > Mn > Cd in brains. Concentrations in tissues were higher in the summer months, but seasonal changes were statistically insignificant (*p* ≥ 0.05). According to Principal Component Analysis (PCA), ANOVA, and Pearson correlation analysis, it was statistically determined that Cd, Cr, Cu, Mn, Ni, and Pb, and the other two elements (Fe and Zn), showed similar accumulation characteristics among themselves. According to transfer factor (TF) calculations, it was determined that there was bioaccumulation (TF > 1) in all tissues for all metals throughout the year, but according to hazard coefficient (HQ) values, only Pb was determined to be >1 and carcinogenic. As a result, after the wastewater is treated with different advanced treatment methods and brought to potable water standards, the accumulation of metals and other micropollutants in the tissues of different species of fish should be monitored for many years.

## 1. Introduction

Wastewater originates from domestic, industrial, and agricultural sources. There were biodegradable organic materials, pathogenic microorganisms, pharmaceuticals, natural/artificial hormones, metal salts, nitrate, phosphorus, sulfur, chlorine, and their compounds in domestic wastewater. Industrial wastewater contains pollutants such as heavy metals, dyestuffs, phenols, plasticizers, polychlorinated biphenyls, oils, fats, suspended or colloidal lubricants, sulfur compounds, sodium chloride, acids, and calcium chloride [1]. Agricultural wastewater contains chemical fertilizers and pesticides along with natural organic matter [2]. Urban wastewater is defined as domestic wastewater or the mixture of domestic wastewater with industrial wastewater and/or runoff rainwater. It may also contain small amounts of agricultural wastewater. So, urban wastewater contains a wide variety of pollutants from domestic and industrial wastewaters [3]. With population growth, the pollution loads carried by urban wastewater increase, water resources become more polluted, and the reduction in freshwater quantities due to climate change, the evaluation of advanced treated wastewater as a new water source will become even more important in the future [4]. After the water consumed as drinking/utility water was transformed into wastewater, it could be brought to a suitable water quality for different reuse alternatives with the second, third, or advanced treatment stages [5]. Although there are many studies on the reuse possibilities of wastewater in different areas, there are not many studies on aquaculture [6]. The relationships between aquaculture wastewater reuse practices and the bioaccumulation of resulting pollutants are poorly understood. Studies have shown that the accumulation of industrial chemicals in wastewater-fed fish varies depending on the species of fish and the chemicals used, and that while low levels are found in muscle tissue, they accumulate in brain tissue [7].

Although there are many pollutants in urban wastewater, heavy metals are among the most common pollutants [8]. The density of heavy metals, which accumulate in tissues of fish and other aquatic creatures in different ways, increases when transferred to an upper ring [9]. High concentrations of metals can lead to genetic alteration, changes in species diversity, growth, physiology and biochemistry, behavior, metabolism, and other processes of the aquatic organisms. Heavy metals disrupt the functions of multiple systems, such as the endocrine and central nervous systems, resulting in various diseases and even death in the early embryonic and larval development stages of fish. Also, long-term exposure to heavy metals reduces the phagocytic capacity of body cells in adult fish and impairs the immune system. Heavy metals disrupt the nervous system in aquatic organisms [10] and are found in higher concentrations in the skeleton than in target organs such as the gills and liver [11]. Metal accumulation can also occur in skin tissue due to direct contact [12]. Heavy metals carried to the human body affect more than one organ and system, and cause cancer and many other diseases [13].

Metal bioaccumulations in the fish tissues are determined using the transfer factor (TF) and bio-concentration factor (BCF). The risk assessment for human health is evaluated with the equations of daily intake (EDI) and hazard quotient (HQ) [14]. In some studies, heavy metals (Pb, Cd, Hg, and As) were investigated in the edible parts (muscle tissue) of some fish species fed in the effluent of wastewater treatment plants [15,16]. The metals (Ag, Al, As, and B) in treated effluent (secondary treatment) and skeleton, skin, eyes, and brain tissues of fish were examined seasonally. HQ (hazard quotient) of Al in all tissues had carcinogenic risk levels [5,17].

Within the scope of this study, metal concentrations (Cd, Cr, Cu, Fe, Mn, Ni, Pb, and Zn) in the skeleton, skin, eye, and brain tissues of *Carassius gibelio* fish fed with the effluent of the Bursa Water and Sewerage Administration Eastern Wastewater Treatment Facility were determined monthly during the winter and summer seasons. The differences in the concentrations between months and different tissues, and the metal’s relationship within the tissues and in wastewater, were investigated statistically. Bioaccumulation of the metals in fish was investigated.

This study aims to measure metal uptake in different organs of a “natural” model—an invasive fish species exposed to a complex and environmentally realistic mixture (treated wastewater). Previous studies have examined metal accumulation in muscle, liver, and gill tissues in fish, but there was no publication about metal accumulation and health risks in the skeleton, skin, eye, and brain tissues of the *Carassius gibelio* species fed in the effluent of advanced treatment plants. Therefore, it represents a scientific novelty. The relevance of this research lies in the growing need to recycle treated wastewater in the context of increasing water scarcity and climate change.

## 2. Materials and Methods

### 2.1. Study Area

**Figure 1:**
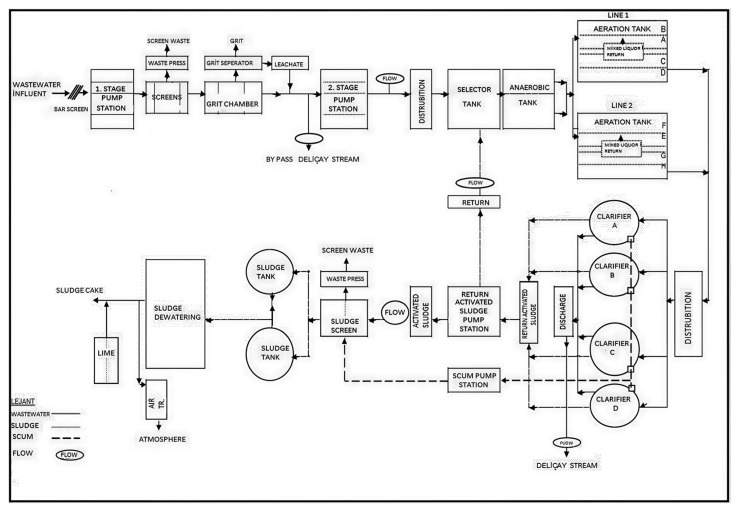
The flowchart of the East Wastewater Treatment Plant.

Bursa City is the 4th largest city in Turkey, located in the Southern Marmara Region and in the Susurluk basin. It is located between 39°35′–40°40′ north latitudes and 28°10′–30°00′ east longitudes. The East Wastewater Treatment Plant treats urban wastewater belonging to the eastern region of Bursa. The project volume of flow is 320,000 m^3^/day for 2030. The 5-stage Bardenpho process, which removed nitrogen–phosphorus, was applied to the facility with advanced biological treatment. At the Eastern Wastewater Treatment Plant of Bursa City, a transmission line was laid from the collection point, where the treated wastewater from the final sedimentation pools was collected and discharged into the stream, to the fish breeding pond. In the effluent of this facility (treated wastewater), the fish of the *Carassius gibelio* species released under human supervision were fed for research purposes [18]. The flowchart of the East Wastewater Treatment Plant was shown in Figure 1.

### 2.2. Metal Measurements of Treated Wastewater and Fish Tissues

Eight metals, including Cd, Cr, Cu, Fe, Mn, Ni, Pb, and Zn, were seasonally (winter and summer) analyzed in TWW (treated wastewater) and the skeleton, skin, eye, and brain tissues of the fish. The studies showed that due to global warming, the summer season was getting longer and the spring and autumn seasons were getting shorter. Therefore, since the effects of summer and winter were more noticeable, sampling in this study was done during the summer and winter months [19]. Cd, Cr, Cu, Ni, Pb, and Zn originate from the metal–chemical, electroplating, and battery industries. Cd, Cu, and Pb could also come from pesticides and fertilizers [10]. While Fe and Mn are naturally present in rocks, mining could cause them to reach high concentrations [20]. Bursa has highly developed metal–chemical paint, automotive, and textile industries, as well as agricultural production. Furthermore, the city has numerous mineral deposits [21]. Therefore, these metals were selected for this study. A total of 20 fish were caught for the monitoring. A total of 5 samples were taken for each season throughout the year, and 3 replicates for each element were made [22]. Invasive species *Carassius gibelio* could adapt to all climate and environmental conditions. Therefore, it spread in Turkey and in many different countries [23]. For these reasons, these fish were fed in the wastewater treatment plant, and this species was chosen for the study.

Wastewater samples were taken in 1 L dark-colored PE bottles using a composite sampling device (Aquacell P2-COMPACT-Aquamatic Co. Ltd., Manchester, UK) [24]. After sampling, wastewater samples were filtered through a Millipore filter (0.45 μm pore-size) and acidified with 0.2% (*v*/*v*) concentrated HNO_3_ in glass bottles, and washed away with nitric acid and Mili-Q water [25].

Freshly dead fish were collected by the basket method, and wastewater samples were taken from the fish breeding pool immediately after the last precipitation at the same time. Experiments have been conducted on dead animals for the purposes of animal welfare, improving production conditions for agricultural breeding, and protecting the natural environment. According to Article 8-k of the Regulation on Working Procedures and Principles of Animal Experimentation Ethics Committees (HAYDEK) of the Ministry of Forestry and Water Affairs, Ethics Committee approval is not required for experiments conducted on dead animal tissue [26]. The fish were carried to the laboratory in the polyethylene caps, and their lengths and weights were registered. The fish were cut from their backbone with the help of a stainless steel knife, and 0.5 g (wet weight) of skeleton, skin, eyes, and brain tissues were separated, and their wet weights were measured. Then, the tissues were fixed to a dry weight by putting them in the drying oven for 24 h. The reason for measuring by dry weight was that wet samples lost moisture even in a short time, leading to measurement errors. The concentrations for both weights were determined using the moisture content of the samples. Afterward, analytically pure 7 mL nitric acid (HNO_3_) and 1 mL hydrogen peroxide (H_2_O_2_) were added to the dried samples placed in pressure-compensated HP500 Teflon caps. The CEM Mars 5 Model microwave device was used to digest the samples. The device operated at 5 psi (1 psi = 6.89 kPa) for 1 min in the 1st stage, at 25 psi for 5 min in the 2nd stage, and at 120 psi for 60 min in the 3rd stage [25,27]. Determination of concentrations was made on samples diluted to 50 mL with ultrapure water. The microwave technique was more reliable and advantageous due to its speed, less risk of contamination, and chemical consumption [28]. European standard reference materials (ERMBB422) and blanks were used in digestion operations for the verification of standardization and calibration. Also, metal concentrations measured in fish of the same species that did not live in wastewater (living in Bursa-Uluabat Lake) were used as a control group for comparison in the analyses. Device readings were made in an ambient of 25 °C. A solution of 5 mg/L prepared from a Merck Mn solution of 500 mg/L was used for the calibration of the device [24]. Metals in all samples (fish and wastewater) were investigated using the VISTA-MPX model of the VARIAN brand’s ICP-OES device (Varian, Pty. Ltd., Victoria, Australia).

### 2.3. Evaluation of Metal Bioaccumulation

Metal concentrations in treated wastewater were evaluated with national and international standards according to usage purposes such as potable, utility, irrigation, and discharge [29,30,31,32,33,34]. Also, the metal concentrations of skeleton, skin, eyes, and brain tissues of the fish samples were interpreted in compliance with national and international standard values [35,36,37,38,39,40,41]. Apart from the evaluation of metal concentrations in fish according to the legislation, they were also interpreted according to statistical calculations. Relationships between treated wastewater and metal concentrations in fish tissues and the metals in the tissues were determined by Principal Component Analysis (PCA) and Pearson correlation analysis. Differences in concentrations between tissues and seasons were determined by factorial ANOVA analysis using the general linear method. The significance level was accepted as 0.05 in all calculations, and the IBM SPSS Statistics 24.0 Program was used [42]. Each concentration reading was made on 3 samples, and all readings were used in statistical calculations [42].

Metal bioaccumulations of the fish tissues were determined using transfer factor (TF) and bio-concentration factor (BCF). The risk assessment for human health was evaluated with the equations of daily intake (EDI) and hazard quotient (HQ).

The transfer factors for fish tissues in aquatic systems were calculated according to Rashed [14] as follows:

TF = M _tissue_ (mg/kg dry weight)/M _sediment or water_ (mg/L) (1)

Bio-concentration factors were determined as follows:

BCF = M _tissue_ (mg/kg wet weight)/M _water_ (mg/L) (2)

In this equation, M _tissue_ was the metal concentration in fish tissue, and M _water_ was the metal concentration in water. In this study, only the water concentrations were used. BCF was already used for the water ambient.

BCF and TF were adversely proportional to concentrations of the exposure ambiance. BCF > 1000 and TF > 1 have been used for hazardous situations in many international legislations. The BCF coefficient was generally used in the evaluation of laboratory studies, and the values determined in the standard for each metal were used in the BCF evaluation [43,44]. Therefore, the combined evaluation of BCF and TF values was more reliable for the assessment of hazards and potential chronic effects. The health risks of consuming the examined tissues of fish samples were determined by calculating the estimated daily intake (EDI) [45,46]. EDI values were calculated using the following equation:

(3) $$EDI=\frac{C_{Fish}\times D_{Fish}}{BW}$$

In this equation, C_fish_: the mean of metal concentration in fish tissue (μg/g dry weight), and D_fish_: the world mean of daily fish consumption (g/day); this value for Turkey was only 1.7 g/day [47], and BW: the mean of human weight (kg). The mean weight of an adult was 70 kg for the USEPA risk analysis evaluation [48]. The hazard quotient (HQ) was calculated by dividing the estimated daily intake (EDI) by the established RfD (reference dose) to assess the health risk from fish consumption [49]. An HQ value of less than 1 indicates no significant health risk [45].

## 3. Results

### 3.1. Evaluation of the Orders of Magnitude of the Seasonal Metal Accumulations in the Tissues

When the orders of magnitude of the metal concentrations in the tissues were evaluated, it was determined that cadmium was in the smallest concentrations in all four tissues. Zinc and iron were found in high concentrations in the skeleton, skin, and eye tissues. Also, iron, zinc, and lead were found to be high in brain tissue. The orders of the metals according to their annual mean concentrations were: Zn > Fe > Mn > Pb > Ni > Cr > Cu > Cd in skeleton; Zn > Fe > Pb > Ni > Cr > Cu > Mn > Cd in skin; Zn > Fe > Cu > Pb > Mn > Ni > Cr > Cd in eye; and Fe > Zn > Pb > Cu > Cr > Ni > Mn > Cd in brain.

In this study, Cu, Fe, and Zn elements, which are biologically useful metals, were found in the highest concentrations in the skeleton, skin, and eye. Also, Cd was found at the lowest level in all tissues, while Pb was found at higher levels than many metals in all tissues.

Within the scope of this study, it was not possible to fully evaluate the degree of toxicity of the metals examined when the concentrations found were evaluated according to national and international standards. Therefore, toxicity levels were evaluated with various coefficients in this study.

Seasonal levels of metals in the brain indicated that the summer concentrations of Cd, Fe, Pb, Ni, Mn, and Zn were higher than their winter concentrations, but the Cr and Cu concentrations of summer were found to be lower than their winter concentrations (*p* ≤ 0.05).

The summer concentrations of Cd, Cr, Cu, Pb, and Zn in the skin were higher than their winter concentrations, unlike metals in brain tissue. Fe, Ni, and Mn concentrations in winter were higher than their summer values (*p* ≤ 0.05).

All metal concentrations of the skeleton in summer were higher than winter concentrations in this study (*p* ≤ 0.05). When the skeleton was compared with other tissues, the metals, except for Zn, were found to be at higher levels in the skeleton and skin.

The highest concentrations of Cd, Cr, Cu, Pb, and Mn were in the skeleton; however, Fe and Ni were highest in the skin, and were 2nd in the skeleton. Zn was highest in the eye, and it was 3rd in the skeleton.

It was determined that the summer values of all metals examined in the eye were higher than the winter values (*p* ≤ 0.05). Seasonal averages of the metal concentrations in the tissues are given in Table 1.

When the orders of magnitude of metal concentrations in tissues were examined seasonally, the seasonal changes in Cd and Cr were generally similar, and their concentrations were found to be the lowest in the eye and the highest in the skeletal tissue. Only in winter, Cd was found to be lower in the brain than in the eye. Similar to Cd and Cr, Cu and Pb were the highest in the skeleton and the lowest in the eye. But during the winter months, Cu and Pb were found to be highest in the brain and skin tissue, respectively. Cd, Cr, Cu, and Pb showed similar levels of accumulation in summer, and according to annual averages, but in winter, higher accumulations were observed in different tissues.

Fe was found at the highest level in the skin throughout the year, compared to other tissues. It was lowest in the brain in the summer, and in the eye tissues in the winter, according to annual average values. Ni concentrations in skin and skeleton were found to be highest in the winter and summer seasons, respectively. Also, it was the highest in skin according to annual averages. Ni was generally found to be lowest in brain and eye tissues. Mn was found to be highest in the skeleton and lowest in the brain and eye tissues in all seasons. Ni and Mn showed similar seasonal changes in terms of accumulation in tissues. Zn was found to be highest in eye tissue and lowest in brain tissue throughout the year.

Zn was found to be highest in the skin after the eye in all months. Zn was similar to Fe in terms of seasonal variations in concentrations in tissues. When the accumulation of metals in tissues was examined generally, it was determined that the accumulations in the skeleton and skin tissue were higher than the other tissues, while the accumulations in the brain and eye tissues were at lower levels.

In this study, it was observed that the annual average concentrations of all metals examined in the skin, except Cu and Zn, were higher than in the eye and brain. In Table 2, the orders of magnitude of seasonal metal accumulations in the tissues were given.

### 3.2. Statistical Analysis

Interactions between metals could also affect accumulation [50]. Therefore, the relationships between metal concentrations in the TWW fed to the fish and the concentrations in the tissues were examined by correlation analysis. It was found that the coefficients of determination (r^2^) of Cr, Cd, Pb, Mn, and Zn calculated between skin tissue and TWW were statistically significant. Studies have shown that the metal accumulation of tissues in direct contact with water (such as skin) was higher than in muscle tissue [12].

When the differences between the tissues were examined according to two-way ANOVA analysis, it was determined that the differences were significant statistically between the skeleton and the skin for Cr, Mn, and Zn, between the skeleton and the eye for Cr, Cd, Pb, Ni, Mn, and Zn, between the skeleton and the brain for Cr, Cd, Cu, Ni, Mn, and Zn, between the skin and the eye for Cd, Pb, Cu, Ni, between the skin and the brain for Cd, Cu, Ni, and Zn, and between the eye and the brain for Pb and Zn. When the seasonal differences in concentrations in tissues were calculated, two-way ANOVA was calculated separately for each tissue, seasonal differences were found to be statistically significant (*p* ≤ 0.05) for the metals generally. However, when the same calculation was performed considering all tissues, seasonal differences were found not to be significant (*p* ≥ 0.05). Therefore, it was observed that biological accumulation for the whole body of the fish continues throughout the year. Correlation and two-way ANOVA analysis results were given in Table 3.

**Figure 2:**
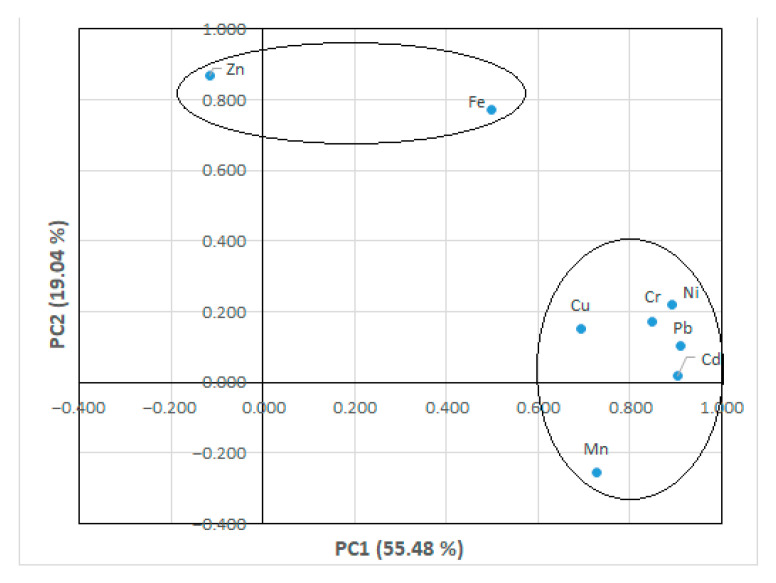
Component plot in the rotated space of PCA.

Multivariate statistical methods could be used to understand the source of pollution or which pollutants come from a similar pollutant source [51,52]. Statistical evaluation could be a method used to find out what to do to take precautions to reduce ecological risks and pollution [53]. PCA was performed within the scope of this study. In PCA analysis, component weights > 0.8 denote a strong effect, and those between 0.5 and 0.8 refer to medium effects [54]. PCA analysis results for the metals in all tissues were given in Table 3. Also, the component plot in rotated space was presented in Figure 2. According to rotation sums of squared loadings, PC1 and PC2 components explained 55.48% and 19.04% of the total variance, respectively. The highest component weight in PC1 was Pb (0.912). After that, Cd (0.906), Ni (0.891), Cr (0.849), Mn (0.727), and Cu (0.693) were obtained. It was thought that the metals found to be significant in PC 1 (Pb, Cd, Ni, and Cr) showed similar accumulation characteristics. Component weights in PC2, which were statistically significant, were found as Fe (0.771) and Zn (0.867).

It was predicted that Fe and Zn in PC2 might have shown similar accumulation characteristics. It could also be said that these metals might originate from wastewater with similar properties. In a study conducted in Bursa on the possible use of different-origin sewage sludge for agricultural purposes, it was determined that plants had accumulations of Pb, Cd, Ni, Cr, Mn, and Cu at levels close to the limit values, unlike the other metals examined. In the same study, as a result of the application of sludge taken from the treatment plant (BWSA) to plants, Zn values were found to be above the limit values [55]. In studies on the accumulation of wastewater treatment sludge in ornamental plants, it was found that the accumulation of Pb, Cd, Ni, Cr, Mn, Cu, Fe, and Zn elements varied according to the plant species [56]. In this study, the accumulation differences in the tissues of metals found to be statistically significant in PC1 and PC2, other than Fe, were also found to be statistically significant according to ANOVA analysis (Table 3). It was observed that most of these metals had statistically significant relationships between wastewater and skin tissue. These findings showed that different statistical evaluations supported each other. The total variance explained by PCA is given in Table 3, and the component plot in rotated space of PCA analysis is shown in Figure 2.

The relationships among the metals in the tissues were examined using Pearson correlation analysis. Accordingly, it was observed that the negative relationships of metals with each other were not statistically significant, but in general, the positive relationships of many metals were significant. Correlation coefficient values r among some metals (Cd and Ni (r: 0.802), Cd and Pb (r: 0.808), Cr and Ni (r: 0.771), Cr and Pb (r: 0.754), and Pb and Ni (r: 0.892)) were found above 0.7 and are statistically significant (*p* ≤ 0.05).

Additionally, the r value between Fe and Zn was 0.453, which was found to be statistically significant (*p* ≤ 0.05). It was thought that metals that were positively correlated with each other might come from similar pollution sources and show similar characteristics in terms of accumulation in fish. The results of Pearson correlation analysis and PCA (PC1 and PC2) among the same metals were found to be statistically significant. The results supported each other.

### 3.3. Determination of Metal Bioaccumulation and Human Health Risks

Values of TF, BCF, EDI, and HQ factors were calculated seasonally. When the seasonal variations in TF values were examined, the bioaccumulation of all metals (TF > 1) in all tissues were found. It was found that summer values were higher than winter values for all metals in the skeleton and eye tissues. The summer values of Cr and Mn in the skin were lower than in winter; other metals were higher.

In brain tissue, it was observed that Cr, Mn, Cu, and Zn were lower in summer, while other metals were higher in summer compared to winter. Accumulations for most metals were found to be more dangerous in the summer months. It was observed that accumulation in the brain (for Cr, Mn, Cu, and Zn) and skin (for Cr and Mn) might have potentially negative effects in the winter.

The TF values of Cr, Cd, Ni, Fe, and Zn elements had the same order of magnitude in the summer and winter. The order of magnitude of the TF values of Pb, Cu, and Mn in tissues varied seasonally.

According to annual averages, the orders of magnitude of TF and BCF values in tissues were found to be similar to the orders of magnitude of concentrations. Nevertheless, it was determined that the seasonal changes in the orders of magnitude of metal concentrations in tissues were different from the TF values. This situation showed that it might be more appropriate to investigate metal accumulation not only according to concentrations but also according to TF values.

When the HQ values were examined in the order of magnitude in tissues, it was observed that they followed the same order in the skeleton, skin, and brain: Pb > Ni > Cr > Cd > Zn > Fe > Cu > Mn. In the eye, the order was Pb > Zn > Ni > Cr > Cd > Fe > Cu > Mn. The lowest HQ level was observed in Mn in all tissues. Except for Pb, the metals were not close to 1, and therefore, they did not pose a definite health risk. However, the fact that the TF values of metals were greater than 1 indicated that they caused bioaccumulation and might have potentially negative effects for children and some sensitive groups. Pb, which was found to be >1 in all tissues, had its highest value in the skin and the second high concentration in the brain. Seasonal and annual averages of TF, BCF, EDI, and HQ values in the tissues are given in Table 4.

## 4. Discussion

### 4.1. Evaluation of the Metal Concentrations in TWW and Fish Tissues According to National and International Standards

Eight metal (Cd, Cr, Cu, Fe, Mn, Ni, Pb, and Zn) concentrations in TWW (treated wastewater) and the skeleton, skin, eye, and brain tissues of the fish were evaluated seasonally (winter and summer) and were compared with national and international standards.

When the TWW that the fish were fed was interpreted by national and international legislations, according to seasonal and annual averages, all metals were found to be lower than the Turkish aquaculture legislation values [29]. According to Turkish, USEPA, and WHO potable water standards, all average values of Cd, Fe, Ni, and Pb were found to be higher than the standards. It was determined that seasonal and annual average values of Cr, Cu, Mn, and Zn were lower than potable water standards [30,34]. When evaluated according to Turkish irrigation water standards, it was found that all average values of Cr, Cu, Fe, Mn, Ni, and Zn were low, while Cd was high. On the other hand, Pb was determined to be higher than the 2nd class (irrigation water) criteria for inland water resources of Turkey [32]. In terms of the parameters examined in this study, it was determined that treated wastewater was not suitable for potable and irrigation water, but was suitable for aquaculture. The reason for this is that aquaculture standards are higher than the drinking and irrigation water standards in terms of the metals examined.

Annual average concentrations of the metals in the fish tissues were evaluated according to the FAO (Food and Agriculture Organization), the WHO (World Health Organization), the US Food and Drug Administration (USFDA), the National Oceanic and Atmospheric Administration (NOAA), the EC (European Union), and the TFC (Turkish Food Codex) [35,36,37,38,40,41,57].

Among the metals in the tissues, Cr was evaluated only according to the FAO and WHO standards because it is not included in other standards. Accordingly, it was found below the limit value in the eye and brain, but above the limit value in the skeleton and skin. According to the FAO, WHO, and EC (0.5 mg/kg dw), Cd was found to be high in the skeleton, skin, and brain, and low in the eye. Concentrations in all tissues were found to be higher than the limit value according to the Turkish Food Codex (TFC). Pb concentrations were generally found to be high in all tissues according to the FAO/WHO [38], EC (1 mg/kg dw), and Turkish Food Codex standards. However, according to FAO [36], Pb was found to be low in the eye [38]. Cu concentrations in all tissues were found to be low according to the Turkish Food Codex, FAO/WHO [38], and NOAA (149 mg/kg dw) standards. Ni concentrations in all tissues were found to be lower than the FAO, WHO, and EC (40 mg/kg dw), NOAA (52 mg/kg dw), and USFDA (70 mg/kg dw) standard values. When Fe concentrations were examined, they were low in the skeleton, eye, and brain, and high only in the skin, according to the FAO and WHO standards [38], but according to FAO’s 1983 standards, Fe was found to be high in the skin and brain [36]. The concentrations of the Mn element in all tissues were found to be higher than the FAO and WHO values [38] and lower than the TFC standard. Zn concentrations were found to be high in all tissues according to the FAO/WHO standards, and low in the skeleton and high in the skin, eye, and brain according to the WHO standards. According to the TFC standards, the skeleton and brain were found to have low values, while skin and eye tissues were found to have high values.

When the accumulations in all tissues were evaluated according to the most used FAO, WHO, EC, and TFC national and international standards, it was seen that Pb and Cd values were at high levels in all tissues, while Cr and Fe were high in some tissues and low in some tissues. While the Mn and Zn elements were high in all tissues according to FAO and WHO standards, some tissues were found to be low according to TFC. Cu and Ni were at low values in all tissues compared to the international standards. When evaluated according to these standards, it was understood that Pb and Cd element accumulation was observed in all tissues examined. It has been observed that there are differences in standards for some metals between international and Turkish standards. However, the FAO, WHO, EC, and TFC limit values were found to be at similar levels.

It has been concluded that when evaluating the toxicity of heavy metal accumulations in fish, it might be erroneous to evaluate only according to concentrations; therefore, it would be more accurate to evaluate them together with coefficients expressing biological accumulation, such as TF and HQ. Annual average concentrations of metals in tissues and FAO, WHO, and TFC standard values were given in Table 5.

### 4.2. Comparison of the Metal Concentrations in the Tissues with Different Studies

The metal concentrations examined were compared with those of different studies. When the annual mean of the metal concentrations examined in the brain tissue in this study was evaluated, it was found that Cd, Cr, Cu, Fe, and Mn elements were lower than in other studies [58,59,60,61,62]. Ni was higher than in other studies [62], and Pb and Zn elements were higher than in some studies [59,60,61,62] and lower than some studies [60,62].

According to the analysis, annual means of Cd, Cr, Ni, and Zn metals in the skin tissue were determined to be higher than the values found in the other studies [63]. However, Cu, Fe, and Mn parameters were found to be lower than in the other studies [59,60,61,63,64]. Also, Pb was found among the values in other studies [60,61,63].

When whole bodies of fish from similar families, such as *Cyprinus carpio* and *Carassius auratus*, were investigated (wet weight), Cu was between 1.25 and 1.32 mg/kg, Pb was between 0.0735 and 0.1560 mg/kg, Zn was between 33.30 and 33.80 mg/kg, and Cd was between 0.0133 and 0.0321 mg/kg in another study. These results indicated that Pb was easily accumulated in the muscle and skeleton of *Cyprinus carpio*, with potential ecological risks as a non-essential toxic metal. When the metal accumulations in different organs were evaluated in the same study, Cu, Pb, and Cd values in skeletal tissue were found to be higher than in head and skin tissue, while Zn in the skeleton was found to be higher than in the skin and lower than in head tissue [65].

In this study, when Cu concentrations in the brain were compared with the average value of the whole fish body in different studies, it was seen that the concentration was among the literature’s values, higher than the literature’s values for Pb, and lower than the literature’s values for Zn. In different studies, Pb and Zn concentrations in the whole body of fish were found to be lower than the values in the skin and eyes in this study, while for Cu, they were found to be higher than the values in the skin and eyes in this study. These comparisons showed that fish had very different levels of metal accumulation depending on their tissues, where examining the concentration averages throughout the body could not provide sufficient information [65].

A few studies used fish eyes as target organs in environmental health assessment. Nevertheless, there were not enough studies associating environmental data with accumulation levels in fish eyes [66]. Fish eyes, lacking true eyelids, are always covered by water. Therefore, they are in permanent and direct contact with contaminants dissolved and associated with resuspended sediment particles, thereby emerging as relevant sites of interaction with metals. When this study was compared with a different study [66], Cd concentrations in this study were found to be higher than in the other study, as Zn was lower, and Cu and Pb were found to be between the concentrations found in the other study. When evaluated together with another study [67], the Cd, Cu, and Pb values found in this study were found to be lower than those in the other study, and Zn was found to be among the values in the other study. In different studies, the concentration levels of the metals examined in eye tissue varied. While some metals examined in this study were found to be higher than in some other studies, some of these metals were found to be lower than the concentrations of other studies.

In the other studies, metals in skin tissue were found to be higher than in the other tissues. It was observed in different studies that the accumulation levels of Ni, Mn, Co, Li, Zn, Pb, Cd, and Fe elements in the skin were higher than in the edible muscle tissue of fish [68]. As shown in a different study, relatively high concentrations of metals were measured in the liver, kidneys, and gills as compared to skin and muscles. Skeleton tissues were used as indicators of environmental toxicology. Since skeletons do not decompose in soil, metals in skeletal tissue are examined even in archeological research [11]. In another study, metal accumulations in the skeleton of *Carassius gibelio* were higher than in the skin, proximal intestine, muscle, distal intestine, gills, and scale tissues. Higher lead concentrations in hard tissues (skeleton and scales) than in other tissues were also reported by Rashed [14].

In general, it was observed that the concentrations in skin and skeletal tissues were higher than in other tissues. Skin and skeleton use many essential metals to form connective tissues. Therefore, these tissues tend to accumulate more metals [22]. Additionally, the skin could absorb metals in water through direct contact [12].

In the other studies, it was observed that metal concentrations in the eye and brain were lower than in the skeleton and skin tissue [69]. The spleen, intestine, and liver also accumulated high levels of cadmium, while the caudal muscle and brain accumulated the lowest levels of cadmium [70]. Hong et al. [71] reported that the brain and eyes contribute to the major lipid component of the fish head, and the lipid contents in the brain were higher than those in the eyes. Also, it was known that high-fat content increased metal accumulation [62]. In this study, it was determined that the accumulations of Cd, Cr, Cu, and Pb in the brain were greater than in the eye.

### 4.3. Evaluation of Metal Bioaccumulation and Human Health Risks

**Figure 3:**
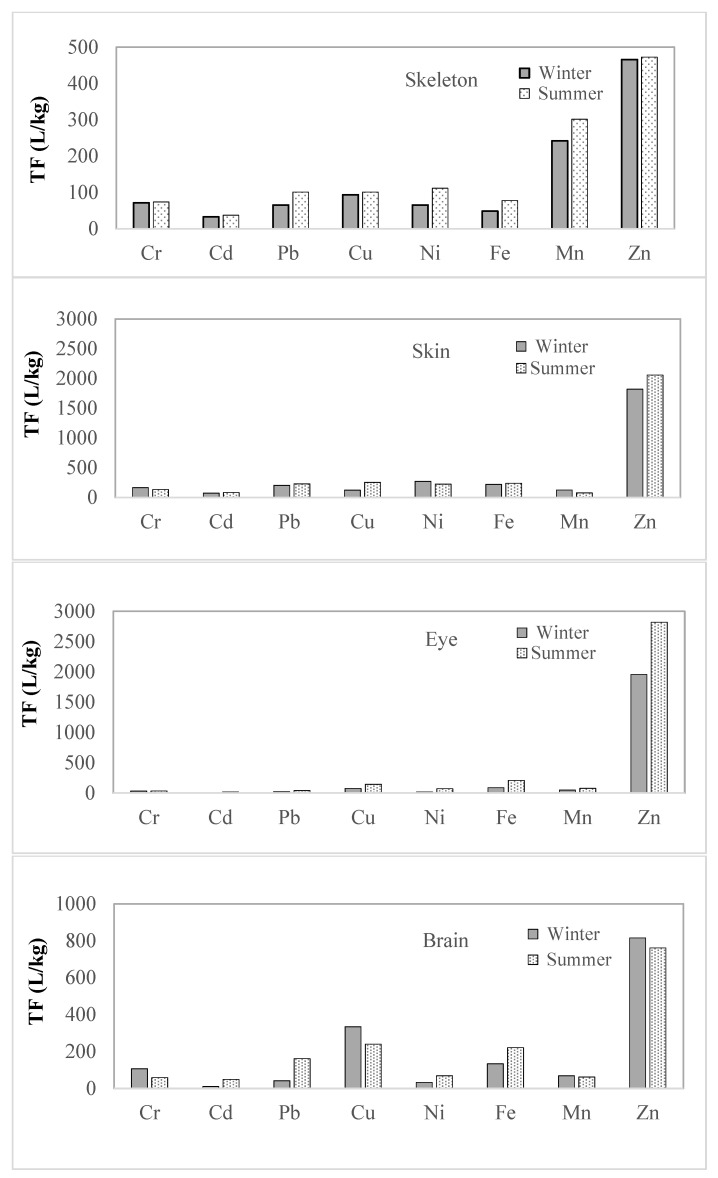
Seasonal TF variations in the metals.

According to the calculations, it was determined that the TF values of all metals were greater than 1, and the BCF values were less than 1000. Since TF values were taken into consideration more than in the scientific literature [14], it was concluded that there was accumulation in every tissue for all metals. The order of annual average TF and BCF values of all metals except Mn was found to be different in the tissues. According to these values, the tissues with the highest accumulations were found in the skeleton and skin, except for Cu and Zn. Cu and Zn accumulations were higher in brain and eye tissues than in skin and skeleton. Fish eyes are highly vulnerable to metals (loids) related to waterborne direct uptake. The eyes signalize better environmental contamination than the brain [72]. Some metals, such as Cu and Zn, could have synergistic effects when accumulated together [73]. When metal accumulations in fish tissues were examined, it was found that the concentrations in the skin and skeleton were lower than in the liver, gills, and kidney, but higher than in the muscle tissue [74]. In previous studies, it was observed that Ag, As, and B elements were found to be higher in the skeleton and skin than in the brain and eyes [17]. Seasonal TF variations in the metals were given in Figure 3. When seasonal variations in TF values were examined, except for Cr and Mn in the skin and Cr, Cu, Mn, and Zn in the brain, other metals were higher in all tissues during the summer. When the seasonal variations in concentrations in tissues were examined, it was observed that, generally, summer values were higher than winter values in all tissues, except for Cr and Cu in the brain, and Fe, Mn, and Ni in the skin, which were not found to be high in winter. Although there were differences in the seasonal variations in concentrations and TF values, they were generally higher in the summer months. This is thought to be due to the acceleration of metabolic activity in fish with the increase in temperatures during the summer months [28].

According to the HQ-Hazard coefficient values, it was seen that the Pb element was higher than 1 in the tissues during the year. Pb was found to be lower than 1 in the eye only in the winter season. When annual averages were examined, it was determined that Pb accumulation was at dangerous levels throughout the year. In different studies conducted on wastewater, it was determined that Pb was in the highest concentrations among the metals examined, and therefore, these wastewaters could not be used for different purposes, and the concentrations of Cu, Mn, Zn, Cd, and Fe elements were below the international standards [75]. In the TWW examined in this study, Cu, Mn, Zn, and Cr were found to be below the potable and irrigation water standards, while Cd and Pb were above these standards [30,31,32,33,34]. When the concentrations in fish tissues were examined, it was found that Cd and Pb were above national and international standards in all tissues [35,36,37,38,41].

In some studies, the bioaccumulation of metals has been shown to be higher in the liver, brain, kidney, and intestines than in muscle tissue. In particular, brain tissue was found to contain higher levels of metals than other organs. Hong et al. [71] reported that the brain and eyes contribute to the main lipid component of the fish head, and the lipid content in the brain is higher than in the eyes. Braune et al. [76] showed that the lipid content of tissues could contribute to the accumulation of contamination in the organs of fish. These results showed that metal bioaccumulation varied according to organs. It was first noticed that it was a health risk for the Chinese because they consumed the heads of fish. The life cycle, ecological needs, and metabolic activities of fish have significant effects on metal levels in their tissues [77]. Direct and continuous exposure to polluted water causes the tissues of fish to accumulate metals more easily than those living in surface waters contaminated by wastewater [78]. Therefore, it is likely that the accumulation in the tissues of the fish studied in this research is higher than in other surface water fish.

Metallic pollution can cause significant oxidative stress in fish, leading to cellular damage and disruption of physiological functions. It also impairs reproduction, leading to decreased fertility and abnormalities. This can reduce population size over time [79].

Consumption of these fish by humans leads to numerous health problems. For example, zinc is important in the metabolism and normal functioning of the human body, but chronic toxicity can lead to the development of hypertension, atherosclerosis, heart disease, and even to chronic liver disease, cirrhosis, or liver cancer, reducing immune function and the levels of high-density lipoproteins. Cd accumulation can lead to varying levels of toxicity. It is classified as a carcinogen for humans, has a long half-life of approximately 30 years, and can cause health problems even at low levels. The substance has also been shown to have harmful effects on the kidneys, skeletal system, and respiratory system [77]. Studies in children have observed many negative effects, such as neurological disorders, kidney damage, and growth disorders [79]. Although TWW complies with the aquaculture standard, the finding that all the metals studied exhibit bioaccumulation suggests that these negative effects may occur in people who consume these fish. Studies on fish include decreased fitness, reproductive interference leading to carcinoma, pathological changes, and ultimately death. It has also been observed that fish exposed to metals show immune system malfunction and thus become vulnerable to infectious diseases [80]. Heavy metals such as cadmium, lead, tin, and chromium have toxic effects even at low concentrations in the tissues of marine animals. However, a few metals (Cu, Fe, Zn, etc.) are of biological importance and exert a toxic effect at very high concentrations.

Although metals such as cadmium and lead have no known role in biological systems [81], Fe, Mn, Cr, Cu, and Zn were found in all plant and animal tissues. The main biological importance of these metals is that they play an active role in lipid, protein, and nucleic acid synthesis, structural and enzymatic reactions, and cell growth, proliferation, and differentiation. Fe is required for the production of red blood cells, but at high concentrations, Fe and Mn could cause pathological events such as iron oxide deposition in Parkinson’s disease [82]. Excess Cu has been associated with liver damage, and Zn might produce adverse nutrient interactions with Cu [83]. Ni assists with enzymes that are needed for the formation of nucleic acids and DNA, but is highly toxic at high concentrations. It could cause gastrointestinal distress, increase red blood cells, and reduce lung function [82].

In fish, Cd causes a decrease in hemoglobin levels and red blood cell count [80]. It was understood that copper reduced fish resistance to diseases, disrupted the structure of tissue cells, and reduced swimming ability and food intake. It was observed that Ni disrupted the gill structure and respiratory mechanism of fish. Pb could cause changes in blood parameters and the nervous system in fish and other animals [84]. Lead negatively affects the nervous, cardiovascular, and hematological systems, skeletal tissue, and kidneys of adults and children. It also causes a decrease in IQ scores, intellectual disability, memory loss, learning difficulties, a decrease in sensory and motor nerve conduction speed, and aggressive and antisocial behavior [13]. In this study, the annual mean concentrations of Cu, Pb, Zn, and Cd in the skeleton were 1.463, 3.533, 43,352, and 0.427 ww mg/kg, respectively, and higher than the whole bodies of fish in the other studies [65].

The statistically related elements Pb, Cd, Ni, and Cr (PC1) originate from wastewater from the paint, battery, and electroplating industries and are in different metal groups in the periodic table. The other related elements, Fe and Zn (PC2), have similar mobility because they are transition metals in the periodic table. Furthermore, both metals are used in the iron and steel industry. These relationships were found not because of similar metal properties but because they come from similar wastewater sources. However, the fact that both Fe and Zn are essential elements for fish tissues also played a role in the strong evidence of this relationship [10]. Cd and Pb levels, which were found to be higher than standard values in wastewater and fish tissues, were statistically correlated with the PC1 group.

In previous studies conducted on the same tissues in fish of the genus *Carassius gibelio*, it was determined that Al accumulation was greater than 1 in terms of HQ value and above carcinogenic levels [17]. When the metal concentrations in the tissues were evaluated according to national and different international standard values, it was seen that, except for Cd and Pb, other metals’ levels varied; some exceeded regulatory limits. According to TF values, it was observed that all metals caused bioaccumulation. For the Pb element, HQ calculations and evaluations according to international standards were found to be consistent with each other, unlike the other metals. Some studies showed that children, pregnant women, and people with weakened immune systems may be more susceptible to the chronic effects of low levels of metal concentrations. For example, developmental neurotoxicity caused by metals or cognitive impairment caused by lead can occur at low concentrations in these sensitive groups. Therefore, even if the overall risk is considered low for the other metals, caution and health warnings are necessary for these sensitive subgroups [79].

## 5. Conclusions

The combined use of bioaccumulation and risk coefficients such as TF and HQ, along with concentrations, allowed for a more accurate assessment, and it was found that the metals bioaccumulated in all tissues. Cu and Zn accumulated more in the brain and eyes, while the others (Cd, Cr, Fe, Mn, Ni, and Pb) accumulated more in the skeleton and skin. Increased metabolic activity during the summer months led to even greater accumulations. It was determined that the metal levels, especially Pb, in wastewater exceeding drinking and utility water standards, were found to be above national and international standards in fish tissues, and that consumption of fish tissues posed a carcinogenic risk and exceeded the carcinogenic threshold (HQ > 1).

Distinctive studies should be conducted on the bioaccumulation of metals in various fish species when wastewater treated using different advanced treatment methods is brought up to drinking water standards. This study will help transform complex contamination data into meaningful public health information, contributing to policymakers and health authorities by establishing safety standards and proactive risk management.

## 6. Additional Requirements

This study complies with ethical standards in the treatment of aquatic organisms. All fish sampling and tissue analyses were performed in accordance with local and institutional guidelines. The wastewater used in the experimental setup underwent secondary treatment and met Turkish aquaculture standards. No human participants or vertebrate animals were directly experimented upon in a manner requiring additional ethical review.

## Figures and Tables

**Table 1:** Seasonal averages of the metal concentrations in the tissues.

Metals	Skeleton (mg/kg ww)		Skin (mg/kg ww)		Eye (mg/kg ww)		Brain (mg/kg ww)	
	Winter	Summer	Winter	Summer	Winter	Summer	Winter	Summer
Cd	0.39 ± 0.0243	0.465 ± 0.241	0.325 ± 0.0939	0.39 ± 0.0585	0.0459 ± 0.0247	0.086 ± 0.0711	0.0358 ± 0.0127	0.177 ± 0.0982
Cr	1.253 ± 0.266	2.149 ± 2.153	1.077 ± 0.0323	1.449 ± 0.062	0.213 ± 0.0907	0.384 ± 0.248	0.536 ± 0.152	0.488 ± 0.376
Cu	1.25 ± 0.143	1.676 ± 0.845	0.613 ± 0.503	1.567 ± 1.102	0.375 ± 0.100	0.898 ± 0.491	1.277 ± 0.084	1.144 ± 0.265
Fe	23.021 ± 1.554	30.227 ± 13.780	38.954 ± 5.160	34.735 ± 2.574	15.954 ± 0.717	30.328 ± 13.77	18.276 ± 0.369	24.696 ± 12.939
Mn	3.858 ± 0.967	6.472 ± 3.439	0.743 ± 0.209	0.608 ± 0.158	0.292 ± 0.0001	0.623 ± 0.306	0.314 ± 0.071	0.38 ± 0.279
Ni	1.926 ± 0.140	2.66 ± 1.547	2.963 ± 0.149	1.991 ± 0.0284	0.177 ± 0.064	0.635 ± 0.190	0.279 ± 0.031	0.472 ± 0.285
Pb	2.898 ± 1.145	4.169 ± 1.460	3.352 ± 0.204	3.501 ± 0.112	0.347 ± 0.234	0.645 ± 0.229	0.527 ± 0.281	1.913 ± 1.202
Zn	40.113 ± 1.385	46.592 ± 16.401	58.247 ± 10.038	75.391 ± 9.625	62.979 ± 9.829	104.002 ± 55.615	20.103 ± 3.359	21.485 ± 3.691

**Table 2:** The orders of magnitude of seasonal metal accumulations in the tissues.

	Winter	Summer	Annual Mean
Cd	Skeleton > skin > eye > brain	Skeleton > skin > brain > eye	Skeleton > skin > brain > eye
Cr	Skeleton > skin > brain > eye	Skeleton > skin > brain > eye	Skeleton > skin > brain > eye
Cu	Brain > skeleton > skin > eye	Skeleton > skin > brain > eye	Skeleton > brain > skin > eye
Fe	Skin > skeleton > brain > eye	Skin > eye > skeleton > brain	Skin > skeleton > eye > brain
Pb	Skin > skeleton > brain > eye	Skeleton > skin > brain > eye	Skeleton > skin > brain > eye
Ni	Skin > skeleton > brain > eye	Skeleton > skin > eye > brain	Skin > skeleton > eye > brain
Mn	Skeleton > skin > brain > eye	Skeleton > eye > skin > brain	Skeleton > skin > eye > brain
Zn	Eye > skin > skeleton > brain	Eye > skin > skeleton > brain	Eye > skin > skeleton > brain

**Table 3:** The results of Pearson correlation, two-way ANOVA, and PCA.

**r^2^ Coefficient of Determination Values Between Concentrations in Wastewater and Tissues**									
		**Cr**	**Cd**	**Pb**	**Cu**	**Ni**	**Fe**	**Mn**	**Zn**
Skeleton	r^2^	0.1816	0.0054	0.2793	0.8211	0.2174	0.2706	0.2901	0.1083
	p	0.6494	0.1194	0.1117	0.2853	0.0679	0.1036	0.2074	0.0718
Skin	r^2^	**0.7964**	**0.6460**	**0.7578**	0.1825	0.0019	0.6489	**0.5933**	**0.7788**
	p	**0.0476**	**0.0326**	**0.0185**	0.2930	0.1004	0.0819	**0.0176**	**0.0300**
Eye	r^2^	0.1808	0.5314	0.0117	0.5327	0.2550	0.0247	0.4798	0.4030
	p	0.9403	0.8553	0.1378	0.4856	0.1188	0.3285	0.5849	0.9413
Brain	r^2^	0.0148	0.0720	**0.6596**	0.1830	0.3471	0.1691	0.2482	0.2291
	p	0.3612	0.4782	**0.0424**	0.1702	0.0750	0.1757	0.6602	0.0663
**Concentration Differences Between Tissues: Analysis of Variance—ANOVA *p* values**									
		**Cr**	**Cd**	**Pb**	**Cu**	**Ni**	**Fe**	**Mn**	**Zn**
Skeleton–Skin		**0.0016**	0.4869	0.2078	0.1653	0.2481	0.1153	**0.0001**	**0.0406**
Skeleton–Eye		**0.0009**	**0.0153**	**0.0028**	0.2467	**0.0099**	0.3976	**0.0002**	**0.0020**
Skeleton–Brain		**0.0032**	**0.0449**	0.2908	**0.0214**	**0.0024**	0.4561	**0.0001**	**0.0500**
Skin–Eye		0.3850	**0.0163**	**0.0146**	**0.0493**	**0.0363**	0.0761	0.2371	0.0697
Skin–Brain		0.3669	**0.0476**	0.3932	**0.0030**	**0.0097**	0.1362	0.3445	**0.0014**
Eye–Brain		0.2649	0.2819	**0.0086**	0.0733	0.2453	0.3561	0.3740	**0.0001**
**Total Variance Explained of PCA Results**									
**Initial Eigenvalues**				**Extraction Sums of Squared** **Loadings**			**Rotation Sums of Squared** **Loadings**		
**Component**	**Total**	**Variance** **%**	**Cumulative %**	**Total**	**Variance %**	**Cumulative %**	**Total**	**Variance %**	**Cumulative %**
1	4.58	57.25	57.25	4.58	57.25	57.25	4.44	55.48	55.48
2	1.38	17.27	74.52	1.38	17.27	74.52	1.52	19.04	74.52
3	0.76	9.54	84.06						
4	0.55	6.85	90.91						
5	0.32	3.96	94.87						
6	0.19	2.33	97.20						
7	0.14	1.79	98.99						
8	0.08	1.01	100.00						

**Table 4:** Seasonal and annual average of TF, BCF, EDI, and HQ values in the tissues.

	Metals	Treated Waste Water (mg/L)	US EPA [44] BCF (L/kg)	RfD (μg.kg bw/Day) USEPA, [49]	Skeleton				Skin				Eye				Brain			
					TF (L/kg)	BCF (L/kg)	EDI (μg.kg bw/Day)	HQ	TF (L/kg)	BCF (L/kg)	EDI (μg.kg bw/Day)	HQ	TF (L/kg)	BCF (L/kg)	EDI (μg.kg bw /Day)	HQ	TF (L/kg)	BCF (L/kg)	EDI (μg.kg bw /Day)	HQ
Winter	Cr	0.0252	19	3	71.05	49.72	0.0435	0.0145	164.21	42.74	0.1005	0.0335	32.29	8.45	0.0198	0.0066	106.24	21.27	0.065	0.0217
	Cd	0.0171	907	1	32.57	22.81	0.0135	0.0135	72.98	19.01	0.0303	0.0303	10.25	2.68	0.0043	0.0043	10.46	2.09	0.0043	0.0043
	Pb	0.0635	0.09	0.05	65.21	45.64	0.1006	2.0113	202.85	52.79	0.3128	6.2565	20.88	5.46	0.0322	0.644	41.45	8.3	0.0639	1.2784
	Cu	0.0191	710	40	93.51	65.45	0.0434	0.0011	123.3	32.09	0.0572	0.0014	75.02	19.63	0.0348	0.0009	333.96	66.86	0.1549	0.0039
	Ni	0.0423	78	1.5	65.06	45.53	0.0668	0.0446	269.17	70.05	0.2765	0.1843	15.99	4.18	0.0164	0.0109	32.95	6.6	0.0338	0.0226
	Fe	0.6835	-	700	48.13	33.68	0.7989	0.0011	219.02	56.99	3.6356	0.0052	89.19	23.34	1.4805	0.0021	133.56	26.74	2.217	0.0032
	Mn	0.0228	-	140	241.8	169.21	0.1339	0.001	125.23	32.59	0.0693	0.0005	48.93	12.81	0.0271	0.0002	68.77	13.77	0.0381	0.0003
	Zn	0.1231	2059	300	465.65	325.86	1.3921	0.0046	1818.38	473.17	5.4362	0.0181	1954.86	511.61	5.8442	0.0195	815.71	163.31	2.4386	0.0081
Summer	Cr	0.0419	19	3	73.27	51.29	0.0746	0.0249	132.9	34.58	0.1352	0.0451	35.01	9.16	0.0356	0.0119	58.18	11.65	0.0592	0.0197
	Cd	0.018	907	1	36.89	25.83	0.0161	0.0161	83.22	21.67	0.0364	0.0364	18.26	4.78	0.008	0.008	49.11	9.83	0.0215	0.0215
	Pb	0.0592	0.09	0.05	100.63	70.42	0.1447	2.8934	227.26	59.14	0.3267	6.5348	41.63	10.9	0.0599	1.397	161.4	32.31	0.2321	4.641
	Cu	0.0238	710	40	100.63	70.42	0.0582	0.0015	253.02	65.84	0.1462	0.0037	144.16	37.73	0.0833	0.0021	240.08	48.07	0.1388	0.0035
	Ni	0.0342	78	1.5	111.14	77.78	0.0923	0.0615	223.71	58.22	0.1858	0.1239	70.94	18.57	0.0589	0.0393	68.94	13.8	0.0573	0.0382
	Fe	0.559	-	700	77.27	54.07	1.049	0.0015	238.79	62.14	3.2418	0.0046	207.3	54.25	2.8143	0.004	220.67	44.18	2.9958	0.0043
	Mn	0.0307	-	140	301.24	210.81	0.2246	0.0016	76.11	19.8	0.0567	0.0004	77.54	20.29	0.0578	0.0004	61.83	12.38	0.0461	0.0003
	Zn	0.141	2059	300	472.19	330.44	1.6169	0.0054	2054.81	534.69	7.0362	0.0235	2818.38	737.6	9.6509	0.0322	761.11	152.38	2.6063	0.0087
Annual Average	Cr	0.0352	19	3	69.04	48.32	0.059	0.0197	137.88	35.88	0.1179	0.0393	32.4	8.48	0.0277	0.0092	72.65	14.55	0.0621	0.0207
	Cd	0.0176	907	1	34.69	24.29	0.0148	0.0148	78.01	20.31	0.0333	0.0333	14.32	3.75	0.0061	0.0061	30.19	6.05	0.0129	0.0129
	Pb	0.0609	0.09	0.05	82.91	58.02	0.1226	2.4524	216.22	56.26	0.3198	6.3956	31.12	8.14	0.046	1.0925	100.06	20.03	0.1480	2.9597
	Cu	0.0219	710	40	95.46	66.8	0.0508	0.0013	191.25	49.77	0.1017	0.0025	111.05	29.06	0.0591	0.0015	276.09	55.27	0.1468	0.0037
	Ni	0.0375	78	1.5	87.37	61.15	0.0796	0.053	253.83	66.05	0.2312	0.1541	41.37	10.83	0.0377	0.0251	50.02	10.01	0.0456	0.0304
	Fe	0.6091	-	700	62.46	43.71	0.924	0.0013	232.46	60.49	3.4387	0.0049	145.17	37.99	2.1474	0.0031	176.2	35.27	2.6064	0.0037
	Mn	0.0276	-	140	267.41	187.14	0.1792	0.0013	94.05	24.47	0.063	0.0005	63.34	16.58	0.0425	0.0003	62.79	12.57	0.0421	0.0003
	Zn	0.1338	2059	300	463	324.01	1.5045	0.005	1919.17	499.39	6.2362	0.0208	2384.28	623.99	7.7476	0.0258	776.27	155.41	2.5224	0.0084

**Table 5:** Annual average concentrations of the metals in tissues and the FAO, WHO, ROPME, and TFC standard values.

Metals	Skeleton (Annual Mean ± Std mg/kg)		Skin (Annual Mean ± Std mg/kg)		Eye (Annual Mean ± Std mg/kg)		Brain (Annual Mean ± Std mg/kg)		FAO/WHO [36] (mg/kg)	FAO [38] (mg/kg) (dw)	WHO [38] (mg/kg) (dw)	TFC, [41] (mg/kg) (ww)
	dw	ww	dw	ww	dw	ww	dw	ww				
Cd	0.610 ± 0.053	0.427 ± 0.037	1.373 ± 0.125	0.357 ± 0.032	0.2519 ± 0.076	0.0659 ± 0.020	0.531 ± 0.063	0.106 ± 0.352	0.5/1 dw, 0.2 ww	0.5	0.5	0.05
Cr	2.430 ± 0.639	1.701 ± 0.448	4.853 ± 0.715	1.263 ± 0.186	1.140 ± 0.326	0.298 ± 0.085	2.5573 ± 0.759	0.512 ± 0.119	1 ww			
Cu	2.090 ± 0.304	1.463 ± 0.212	4.188 ± 1.833	1.09 ± 0.477	2.4319 ± 0.979	0.636 ± 0.261	6.046 ± 0.419	1.210 ± 0.332	0.03 ww	30	30	20
Fe	38.045 ± 5.148	26.624 ± 3.60	141.593 ± 8.107	36.844 ± 2.109	88.421 ± 27.461	23.141 ± 7.187	107.322 ± 1.843	21.486 ± 16.03	100	180	109	
Mn	7.380 ± 1.867	5.165 ± 1.307	2.595 ± 0.259	0.675 ± 0.067	1.748 ± 0.632	0.457 ± 0.165	1.733 ± 0.354	0.347 ± 0.165	1 dw			20
Ni	3.276 ± 0.524	2.293 ± 0.369	9.518 ± 1.867	2.477 ± 0.485	1.551 ± 0.875	0.406 ± 0.229	1.875 ± 0.154	0.375 ± 0.482	10 dw	55	30	
Pb	5.049 ± 0.908	3.533 ± 0.635	13.167 ± 0.286	3.426 ± 0.074	1.895 ± 0.569	0.496 ± 0.149	6.093 ± 1.403	1.22 ± 3.461	0.5 dw	2	0.5	0.3
Zn	61.95 ± 4.629	43.352 ± 3.23	256.785 ± 32.94	66.819 ± 8.571	319.016 ± 78.37	83.490 ± 20.511	103.865 ± 16.77	20.794 ± 3.453	100 dw, 40 ww			50

## Data Availability

The data presented in this study are available on request from the corresponding author.

## Abbreviations

The following abbreviations are used in this manuscript:

PCA	Principal Component Analysis
TF	Transfer factor
BCF	Bio-concentration factor
HQ	Hazard quotient
